# Denosumab-Induced Recurrent Severe Hypocalcaemia in a Patient With Chronic Kidney Disease: A Case Report

**DOI:** 10.7759/cureus.73476

**Published:** 2024-11-11

**Authors:** Rujina Begum, Summon Mir, Brian Lee

**Affiliations:** 1 Emergency Medicine, Midland Metropolitan University Hospital, Birmingham, GBR; 2 Geriatrics, Midland Metropolitan University Hospital, Birmingham, GBR; 3 Diabetes and Endocrinology, Midland Metropolitan University Hospital, Birmingham, GBR

**Keywords:** chronic kidney disease (ckd), denosumab, drug-induced hypocalcaemia, osteoporosis, severe hypocalcaemia

## Abstract

Denosumab is a human monoclonal immunoglobulin G2 (IgG2) antibody that is used in the management of osteoporosis and various bone-related disorders to help strengthen bones and prevent fractures. Declining renal function and the drug denosumab are both associated with an increased risk of hypocalcaemia. The combination of both factors in a patient, along with the long half-life of the drug, can cause further exacerbation of this risk. A thorough understanding of pharmacokinetics is therefore essential when encountering drug adverse effects. We present a case of recurrent severe hypocalcaemia secondary to denosumab administration in a patient with chronic kidney disease. This case report highlights the importance of close monitoring of calcium levels in patients with established risk factors for hypocalcaemia, as well as in those who have achieved resolution of denosumab-induced hypocalcaemia.

## Introduction

Denosumab is recommended for the primary and secondary prevention of osteoporotic fragility fractures in patients meeting specific criteria. It is administered as a 60 mg subcutaneous injection every six months. A larger dose may be given at four-week intervals for the prevention of skeletal-related events in patients with bone metastasis from solid tumours [1].

Denosumab works by attaching to a protein called receptor activator of nuclear factor kappa beta ligand (RANKL), blocking it from activating its receptor, RANK, found on the surface of osteoclasts and their precursors [2]. This prevents the development of osteoclasts by inhibiting their differentiation, activation and survival, which leads to a reduction in osteoclast-driven bone resorption and turnover.

Denosumab-induced hypocalcaemia is a known adverse effect with the potential to cause life-threatening arrhythmias [3]. The inhibition of osteoclastic bone resorption reduces the efflux of calcium from the bone into the bloodstream. There is no significant effect of renal impairment on denosumab pharmacokinetics [4]. Dose adjustments are therefore not required in patients with chronic kidney disease (CKD). However, several studies have demonstrated that patients with CKD are at increased risk of hypocalcaemia, especially when there is severe impairment of renal function.

## Case presentation

**Figure 1:**
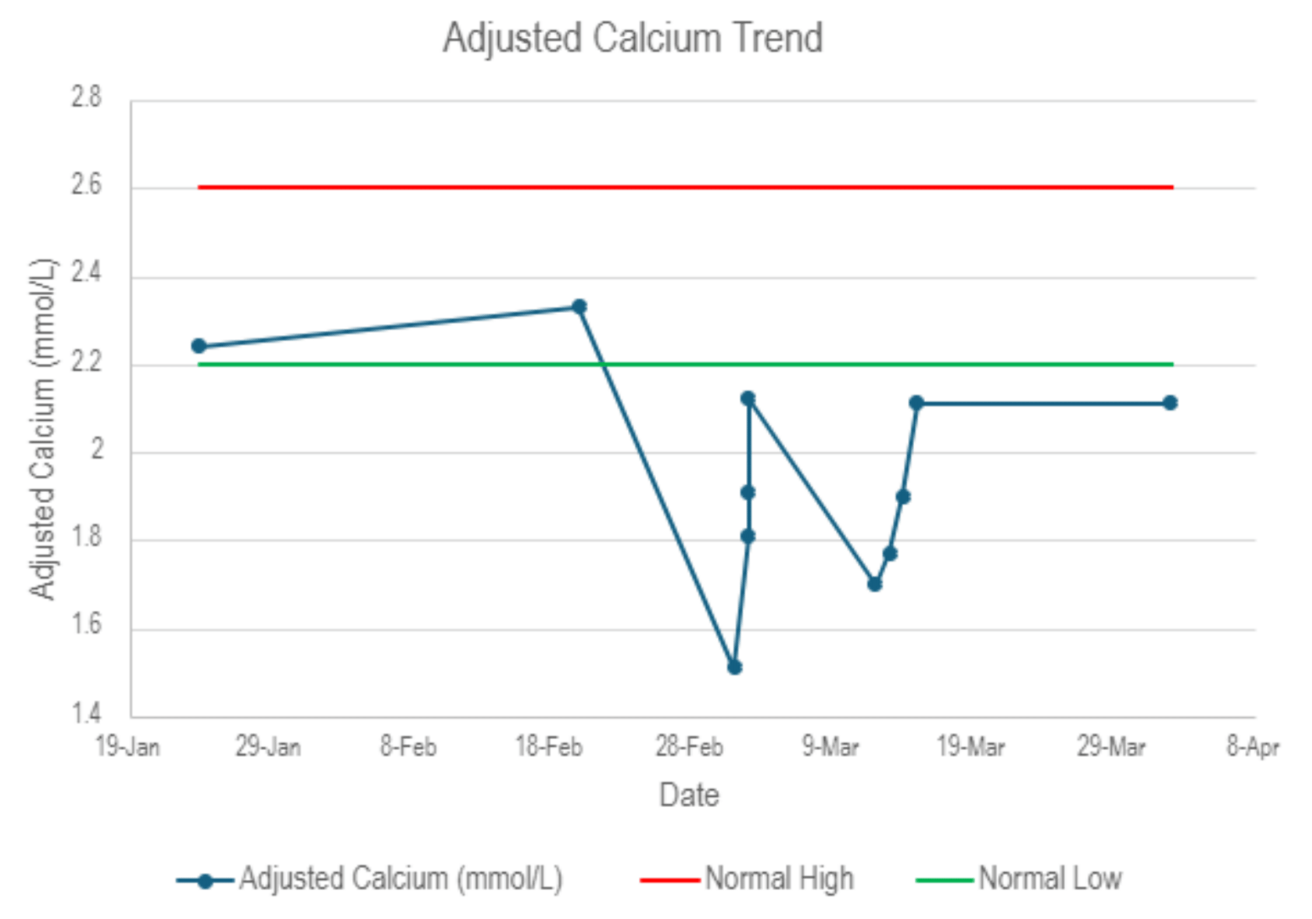
Adjusted calcium levels before, during and after hospital admissions Denosumab was administered on February 23, 2024. The first presentation to hospital with hypocalcaemia was on March 2, 2024. The second presentation to hospital with hypocalcaemia was on March 14, 2024.

A 70-year-old female patient presented to the hospital with a three-day history of dizziness, feeling weak, generalized pain and pins and needles in the face, arms and legs. The patient received her first dose of 60 mg of subcutaneous denosumab for the treatment of osteoporosis eight days prior to the presentation. This is on the background of stage 4 CKD. Other past medical history includes chronic diarrhoea, hypertension, diabetes, chronic obstructive pulmonary disease, B12 deficiency, iron deficiency and coronary artery disease. The examination was negative for Chvostek's sign. Trousseau's sign was not tested for. Blood tests showed adjusted calcium of 1.51 mmol/L (reference range 2.2-2.6 mmol/L). For comparison, adjusted calcium was 2.33 mmol/L two weeks prior to this. Figure 1 shows the trend of adjusted calcium levels before, during and after hospital admissions. Other laboratory results (Table 1) showed a high parathyroid hormone (PTH) level and normal levels of vitamin D, magnesium and phosphate. ECG showed sinus rhythm with a normal QT interval of 441 ms. The patient required both a stat dose and a 24-hour infusion of intravenous calcium gluconate. The patient reported no longer having symptoms during a review one hour after the initiation of therapy. The 6-hour and 12-hour adjusted calcium levels were 1.81 mmol/L and 1.9 mmol/L, respectively, reaching 2.12 mmol/L after the completion of the infusion. The patient was discharged home with oral calcium and vitamin D replacement, with a plan for the general practitioner to repeat blood tests in one week.

**Table 1:** Laboratory results from the initial presentation to hospital eGFR: estimated glomerular filtration rate

Test	Result	Reference Range
Adjusted calcium	1.51 mmol/L	2.2-2.6 mmol/L
Phosphate	1.05 mmol/L	0.8-1.5 mmol/L
Magnesium	0.87 mmol/L	0.7-1 mmol/L
Parathyroid hormone	72.80 pmol/L	2.7-11.1 pmol/L
Vitamin D	61 nmol/L	50.1-220 nmol/L
eGFR	24 mL/min/1.73 m2	≥ 60 mL/min/1.73 m2
Creatinine	185 umol/L	50-98 umol/L

She was referred to the hospital by her general practitioner nine days later for recurrent severe hypocalcaemia, with an adjusted calcium of 1.70 mmol/L. She reported no overt symptoms. The only symptom of note was that she reported feeling more tired than usual. Given severe hypocalcaemia and the need for infusion during the previous admission, we administered an infusion of calcium gluconate along with oral calcium replacement. Six-hourly serum calcium checks were carried out. Repeat blood tests showed an adjusted calcium of 2.11 mmol/L the following day.

She was sent home on oral calcium and vitamin D replacement. Her case was discussed in the osteoporosis multidisciplinary team meeting, and it was concluded that it would be inappropriate to continue with further denosumab injections.

## Discussion

The efficacy of denosumab has been demonstrated in various studies. One such retrospective cohort study involving 483,487 patients found that using denosumab compared with alendronate reduced the risk of major osteoporotic, hip, nonvertebral, non-hip nonvertebral and hospitalised vertebral fractures by 30-50% [5]. However, denosumab is associated with several adverse effects, including hypocalcaemia, hypophosphatemia, osteonecrosis and gastrointestinal disturbance [1].

Current practice involves the correction of hypocalcaemia and low vitamin D prior to the initiation of therapy, with the continuation of supplements during treatment [1]. However, despite these precautions, there have been reports of severe hypocalcaemia with denosumab use [6-8]. Therefore, it is crucial to identify factors that may accentuate this risk. One significant factor to consider in this context is the presence of CKD. The increased risk is likely due to CKD-associated or secondary hyperparathyroidism, as seen in this case, which places greater dependence on PTH-mediated bone resorption for the maintenance of serum calcium.

The original denosumab Fracture Reduction Evaluation of Denosumab in Osteoporosis Every 6 Months (FREEDOM) trial reported that there is no increased risk of hypocalcaemia with denosumab administration [9]. Secondary data analysis conducted by Jamal et al., which used subjects from the FREEDOM trial, found that adverse effects did not differ by the level of kidney function [10]. However, no participants in the study had stage 5 CKD and only 73 out of the nearly 8000 participants in the study had an estimated glomerular filtration rate (eGFR) of 15-29 mL/min. The results from this trial may, therefore, not be widely applicable to those with advanced renal disease. In contrast, a study conducted in the United States reported a higher incidence of hypocalcaemia in patients with severe CKD and kidney failure receiving denosumab compared to those with mild to moderate or normal renal function [11]. Several other studies have drawn similar conclusions [12,13]. However, the exact mechanisms underlying this increased risk have not been comprehensively studied.

What we do know is that patients with advanced CKD are more likely to have concomitant mineral bone disorder. The liver converts vitamin D3 to 25-hydroxyvitamin D3 using the enzyme 25-hydroxylase. 1-alpha hydroxylation occurs in the kidneys, producing calcitriol, the active form of vitamin D. Calcitriol increases bone resorption and intestinal absorption and decreases renal excretion of calcium [14]. Low levels of vitamin D consequently cause hypocalcaemia due to a reduction in the activity of the above processes. In CKD, there is also reduced excretion of phosphate, which forms complexes with calcium, and these complexes are deposited in the vasculature and other tissues. This leads to a reduction in serum calcium. Additionally, there is a compensatory increase in fibroblast growth factor-23 (FIG-23) secondary to phosphate retention. FIG-23 causes reduced production of renal 1α-hydroxylase, and there is evidence to suggest that it also suppresses PTH [15]. Ultimately, these mechanisms disrupt calcium homeostasis. The use of denosumab, which downregulates osteoclast activity, can therefore increase the predisposition to developing hypocalcaemia in these patients.

A notable finding in our patient’s case was the presence of recurrent hypocalcaemia. Our patient presented with a second episode of severe hypocalcaemia nine days after being discharged. Denosumab has a mean half-life of 25.4 days [16]. As a result, patients may experience relapsing or refractory hypocalcaemia [8]. We therefore recommend regular monitoring and surveillance of calcium levels in patients who develop hypocalcaemia, even after its resolution. This close monitoring allowed the recurrent episode in our patient to be recognized and treated before the development of any substantial symptoms or complications. Furthermore, maximal serum concentrations of denosumab are seen in 5-21 days [17]. We thus advise that in patients with known risk factors for hypocalcaemia, calcium levels should be monitored regularly after each dose, with intervals between re-checks being dependent on the patient's individual risk. This is in addition to the existing practice of checking calcium levels prior to each dose in all patients, irrespective of their risk. Additionally, as done in our patient’s case, it would be advisable to consult with the patient’s nephrologist prior to the initiation of denosumab in patients with advanced renal disease.

We also recommend that patients receive thorough counselling regarding the risk of hypocalcaemia. If symptoms do occur, patients should be advised to seek urgent medical attention. These symptoms include but are not limited to muscle twitching, cramps, spasms and paraesthesia of the fingers, toes or perioral region. It is also a good practice to signpost patients to national charities and societies for further information and support.

## Conclusions

There are a limited number of clinical studies exploring the relationship between CKD or other predisposing factors and the risk of hypocalcaemia with denosumab. Further studies are required to draw more accurate conclusions. However, we are able to use the existing evidence to make decisions regarding calcium monitoring. Due to the long half-life of denosumab, we recommend close monitoring of calcium levels even after the resolution of an initial episode of hypocalcaemia. Additionally, we recommend that patients with known risk factors for hypocalcaemia should have regular monitoring of their calcium levels, preferably weekly at first, then re-checked at intervals appropriate to the patient's level of risk.
